# Microbiological Hazard Identification and Exposure Assessment of Poultry Products Sold in Various Localities of Hyderabad, India

**DOI:** 10.1100/2012/736040

**Published:** 2012-04-19

**Authors:** Rao V. Sudershan, R. Naveen Kumar, L. Kashinath, V. Bhaskar, K. Polasa

**Affiliations:** ^1^Food and Drug Toxicology Research Centre, National Institute of Nutrition (Indian Council of Medical Research), Jamai-Osmania, Hyderabad 500007, India; ^2^Statistical Division, National Institute of Nutrition (Indian Council of Medical Research), Jamai-Osmania, Hyderabad 500007, India

## Abstract

A study was carried out to identify microbiological hazards and assess their exposure associated with consumption of poultry based street food served in different localities of Hyderabad. The study indicated that chicken 65, chicken fried rice, chicken noodles, chicken Manchuria and chilly chicken are the most common recipes. A process flow diagram was developed to identify critical control points in the food item. After analysis of the samples at each level of preparation, it was observed that rice and noodles were kept at room temperature for about 5-6 hrs which was a critical control point. A total of 376 samples including chicken fried rice, chicken noodles, boiled noodles and boiled rice were collected from circle 1, 2, 3, 4, 5, 6, and 7 of Greater Hyderabad municipal corporation (GHMC) and analyzed for microbiological examination. The most prevalent pathogenic bacteria isolated were *S. aureus* (3.4 log 10 cfu/g) and *B. cereus* (3.4 log 10 cfu/g). *Salmonella* spp. was present in salads (3.2 log 10 cfu/g) and hand washings of the food handler (3.5 log 10 cfu/g). *Salmonella* contamination was found in salads served along with chicken fried rice and chicken noodles than in the food.

## 1. Introduction

Microbiological risk assessment (MRA) is an emerging tool for the evaluation of the safety of food and water supplies. World Health Organization (WHO) suggests that microbiological risk assessment should be carried out so that appropriate remedial measures can be adopted to curtail the episodes of foodborne illness as a result of consumption of these foods. FAO and WHO have important tasks in developing and standardizing MRA at an international level and informing risk managers at national and international level. The FAO/WHO guidelines on exposure assessment for microbiological hazards in food are part of these activities [1].

Street-vended foods are defined as those foods prepared on the street and ready to eat, or prepared at home and consumed on the street without further preparation [2]. Most handlers of street-vended foods in developing countries are largely ignorant of basic food safety issues. Consequently, street foods are commonly exposed to dangerous abuses, often at all stages of handling. The washing of hands, utensils, and dishes is often done in buckets or bowls [3].

Quality and safety are two common concerns cited with regards to street foods. If the street foods are prepared from high-risk foods like poultry meat, the concern is much more. Contaminated food is a common source of human infections and poultry products are considered as a significant source of infection for humans [4, 5]. In India during recent years, there has been an increasing trend towards the sale and consumption of street foods. This phenomenon is more seen in the urban areas of the country. Street food-vendors are common in urban and semiurban areas, but they also operate in rural areas, particularly if there is a market or community fair [6]. Studies on urban street food in India are few. In India and other developing countries, little information is available regarding the foodborne illnesses due to consumption of street foods; however, the study conducted by Das et al. [7] on street-vended Indian chats sold in Bangalore indicated that food samples revealed high loads of bacterial pathogens such as *S. faecalis, E. coli, S. aureus, Bacillus* spp., *Klebsiella* spp., and *Pseudomonas* spp. The study conducted by Mahale et al. [8] on fresh squeezed juices of sugarcane, lime, and carrot sold by street vendors in Mumbai city indicated that the total viable counts of all samples were approximately log 6.5 cfu/100 mL with significant load of *coliforms, faecal coliforms*, *Vibrio*, and *Staphylococcal *counts.

Various studies have been reported on microbiological quality of street foods in India but the extent of risk associated with the consumption of contaminated street foods depends on the dietary exposure to the contaminant. In view of this an attempt was made to carry out microbiological hazard identification and exposure assessment studies of poultry-based street foods. The aim of this study was to determine the potential microbiological hazards and their exposure associated with the consumption of poultry-based street foods.

## 2. Materials and Methods

### 2.1. Study Area

The study was carried out in Hyderabad which is the capital of Andhra Pradesh, India. As of 2010 it is the sixth most populous city and sixth most populous urban agglomeration in India. The twin cities of Hyderabad and Secunderabad come under the ambit of a single municipal unit, the Greater Hyderabad Municipal Corporation. For administrative purpose, Greater Hyderabad Municipal Corporation has been divided into many circles with each circle being homogeneous within and different from other circles. Random sampling procedure was adopted to select seven circles out of it. The sample required for the study was obtained using proportionate representation according to size.

### 2.2. Questionnaire

Data on food preparation, handling, and storage practices were collected using structured questionnaire that had both observational and responsive questions. Equipment used for the preparation of food and source of water for both utensil cleaning and cooking, utensil cleaning methods and hand washing were considered as food preparation and handling practices while storage of leftover food, storage length were considered as food storage practices. Information on status of the premises, storage conditions for poultry before cooking, cutting and chopping place, status of the serving plate, peeling and cleaning of vegetables, cleanliness of the cloth used (cleaning cloth), cleanliness of clothing (vendor), provision for waste disposal, presence of rodent droppings in the outlet, and exposure to insects were also noted. The questionnaire was pretested in ten street food outlets from the selected circles. Sixty four randomly selected vendors were successfully interviewed.

### 2.3. Hazard Analysis

Hazard analysis critical point (HACCP) is a system which identifies, evaluates, and controls hazards which are significant for food safety. The hazard analysis includes observing food preparation and practices to identify the sources and modes of contamination. Measurement of temperatures of food and near surface areas of foods that were displayed for sale was carried out. Food samples were collected during various stages of preparation and tested for contaminating microorganisms.

### 2.4. Sample Collection and Processing

A total of 376 food samples and 110 samples of hand washings, drinking water, and salads (Onion and Lemon) were collected randomly from 7 circles. The samples consisted of chicken fried rice (recipe made from rice fried in a wok and fried chicken), chicken noodles (recipe made from noodles and chicken fried in a wok), and boiled rice (rice prepared and used for the preparation of chicken fried rice), boiled noodles (noodles prepared and used for the preparation of chicken noodles). Usually street food outlets do business during evening hours. Therefore samples were collected accordingly. Sterile polythene zip bags were used for collection of samples.

Immediately after collection of the sample, temperature of the food sample was noted down. Samples were carried to the laboratory in aseptic condition. The polythene bags with food samples were kept in an ice box maintained at 6–10°C and processed within 2–4 hrs. Twenty-five of each food samples were weighed and transferred to 225 mL of sterile-buffered peptone water. The diluent of buffered peptone water was then inoculated on to the respective media.

### 2.5. Identification and Enumeration

 Identification and enumeration of the bacteria was performed as described by US FDA bacteriological analytical manual. After thorough mixing of the sample in buffered peptone water, the sample was inoculated to selenite broth for *Salmonella* enrichment. 0.1 mL of the sample was inoculated on selective media like XLD (Xylose Lysine Deoxycholate Agar) to detect the presence of *Salmonella*, BPA (Baired parker Agar) for *S. aureus*, and BCA (Bacillus Cereus Agar with egg yolk and polymyxin) for *B. cereus.* After an incubation period at 37°C for 24 hrs, the colonies were observed and the identification of pure culture from food samples was done by studying colony characteristics, microscopy, motility test, and biochemical characteristics. Red colonies with black center on XLD were identified as presumptive *Salmonella* spp. and the confirmation was done by Gram staining, Motility test, carbohydrate fermentation test (Gas from Glucose) Indole test, Methyl Red Voges Proskauer test, and H_2_S production test. Colonies on BPA were identified as presumptive *Staphylococcus *spp., and the confirmation was done by Grams staining and coagulase test. Colonies showing bluish green colour on BCA were identified as *Bacillus cereus*, and the confirmation was done by Gram staining and carbohydrate fermentation test.

### 2.6. HACCP Study

**Figure 1:**
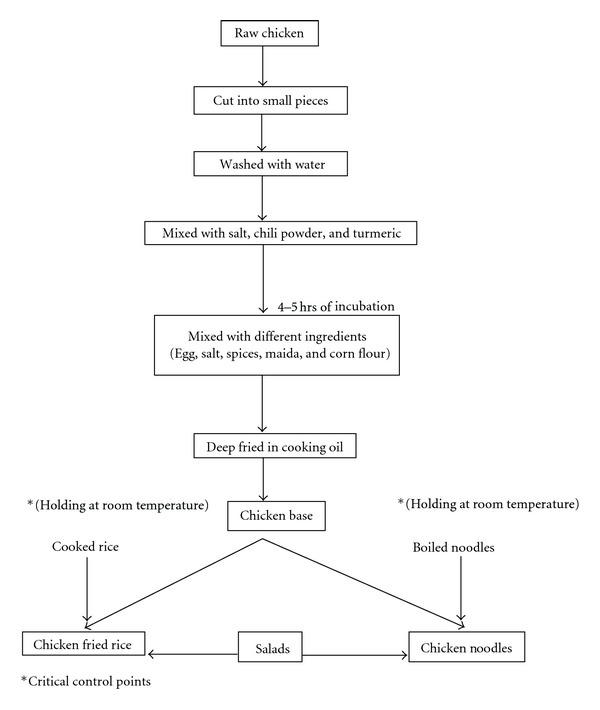
Flow chart to identify hazard analysis and critical control points.

 A total of 35 vendors were interviewed to collect the information on method of preparation of each food items. Study indicated that the common recipes of poultry-based street foods are Chicken 65 (spicy, deep fried chicken, 65 refers to the age of chicken used to prepare this dish), Chicken Fried Rice, Chicken Noodles, Chicken Manchuria (chicken with vegetables in a spicy sauce), chilly chicken (fried chicken with more chillies), and Ginger chicken (fried chicken with more ginger). These food items are the adaptation of Chinese seasoning and cooking techniques to Indian tastes. It is said to have been developed by the small Chinese community that has lived in Kolkata, a capital city of West Bengal, India, for over a century. Flow charts for each food items were prepared. After analysis of the samples at each level of preparation, it was indicated that cooked rice and noodles used for the preparation of chicken fried rice and chicken noodles were kept at room temperature for about 5-6 hrs which was a critical control point. Such kinds of critical control points were not observed in the preparation of Chicken 65, Chicken Manchuria, chilly chicken, and Ginger chicken. Flow chart to identify critical control point in chicken fried rice and a chicken noodle is given in Figure 1.

### 2.7. Statistical Analysis

The analysis was done by descriptive analysis (Mean, Standard deviation, Standard Error, Minimum, and Maximum) for each category of the groups. Differences between the group were tested by nonparametric Kruskal-Wallis ANOVA considering the heterogeneity of variance and individual pair difference was done by Mann-Whitney *U*-test (SPSS 14.8 windows version was used).

## 3. Results

### 3.1. Incidence of Foodborne Pathogens in Food Samples

Sample analysis indicated the presence of one or more of the foodborne pathogens in each food sample. Sixty percent of samples were contaminated with either *B. cereus* or *S. aureus.* The incidence of *S. aureus*, *Salmonella* spp. and *B. cereus* in poultry products sold in various localities of Hyderabad is shown in Table 1. *S. aureus* was isolated in 31% of the food samples and *B. cereus* was detected in 58% of the food samples. Foodborne pathogens were very high in salads and drinking water.* Salmonella *contamination was found more (43%) in salads than in the street food. The salads are served along with the street foods like chicken fried rice and chicken noodles. Since *Salmonella* contamination was more in salads, it is likely that salads contribute to contamination of the street food with *Salmonella*.

### 3.2. Bacterial Counts

 Logarithmic mean ranges and medians of the bacterial counts for the food and other sample categories are shown in Table 2. Pathogen levels in all samples ranged from 2.0 to 4.5 log 10 cfu/g with a mean of log 10 cfu/g, while *S. aureus* concentration ranged from 2 to 4.4 log 10 cfu/g with a mean of 3.4 log 10 cfu/g. *B. cereus* numbers ranged from 2 to 4.4 log 10 cfu/g with a mean of 3.4 log 10 cfu/g and the *Salmonella *spp. concentration 2–4.1 log 10 cfu/g with a mean of 2.8 log 10 cfu/g. It was found that *Salmonella *spp. was present in salads (3.2 log 10 cfu/g) (Table 3) and hand washings of the food handler (3.5 log 10 cfu/g). Statistical analysis demonstrated a significant difference in pathogen concentration among food categories (*P* < 0.001).

### 3.3. Distribution of Food-Borne Pathogens in Different Circles

Statistically significant differences were observed in the levels of contamination in different circles of Hyderabad. The quantum of *B. cereus* load in chicken fried rice collected from circle 1 (*P* < 0.001) was significantly different from circles 2, 3, 4, 5, 6, and 7. The *B. cereus* count was more in circle 1 (4.6 log 10 cfu/g) when compared with circle 2 (1.9 log 10 cfu/g), 3 (3.4 log 10 cfu/g), and 4 (3.1 log 10 cfu/g). Similarly a significant difference was observed in *S. aureus* count isolated from chicken noodles of circles 1 (*P* < 0.01), 3, and 7. The *S. aureus* count was more in circle 1 (3.8 log cfu/g) when compared with circle 3 (2.7 log cfu/g) and it was not detected in circle 7. These findings showed that pathogenic bacterial population is high in circle 1 than in circles 2, 3, 4, 5, 6, and 7, and it is indicated that bacterial population varies from locality to locality.

### 3.4. Exposure Assessment of Food Samples

About twenty-three percent (23.4%) and fifty-six (56.4%) percent chicken fried rice samples were contaminated with of *S. aureus* and *B. cereus*, respectively. Twenty-four (24.5%) and fifty-nine percent (59.6%) chicken noodle samples were contaminated with *S. aureus* and of *B. cereus*, respectively. *Salmonella *spp. was not detected in both of these food samples. The salads served along with chicken fried rice and chicken noodles were contaminated with *S. aureus* (61%), *B. cereus* (48%), and *Salmonella* spp. (43.5%). Initial contamination of the raw material like boiled rice and boiled noodles was analysed and considered for exposure assessment. Thirty-five (35%), fifty-one (51%) and about eight percent (8.5%) boiled rice were contaminated with *S. aureus, B. cereus*, and *Salmonella* spp., respectively. Forty-three (43%), sixty-six (66%) and six percent (6.4%) boiled noodles were contaminated with *S. aureus*, *B. cereus*, and of *Salmonella* spp., respectively. These cooked materials and salads were kept at room temperature for about 4-5 hrs before adding it to the final product. The *Salmonella *spp. was not detected in the final product of food such as chicken fried rice and noodles; however the salads which were served along with these items were heavily contaminated with *Salmonella *making these foods unsafe. Level of sanitation was poor, most (70%) of the street food outlets were located very adjacent to the road, and few (15%) outlets were located near the municipal garbage bins. All the vendors were observed to be cooking the food with bare hands without wearing hand gloves. Very few of the outlets (7%) were using refrigerator to store the poultry before cooking. Most of the (57%) outlets were using wet, unclean serving plates without any protective material like butter paper to serve the food. The exposure assessment studies indicated that the mean weight of the chicken fried rice per serving was 456 g, mean microbial load of *B. cereus* in chicken fried rice is 3.0 × 10^4^ cfu/g, and the mean exposure per serving was 1.3 × 10^7^ cfu. The mean exposure of *S. aureus* per serving was 1.7 × 10^6^ cfu. The mean weight of the chicken noodles per serving was 377 g, and mean exposure of *B.cereus* in Chicken Noodles per serving was 3.7 × 10^6^ cfu. The mean exposure of *S. aureus* in Chicken Noodles per serving was 3.9 × 10^5^ cfu. The mean weight of the salads served along with chicken fried rice and chicken noodle was 19 g. The mean exposure of *S. aureus* per serving was 1.1 × 10^5^ cfu, the mean exposure of *B. cereus* in salads per serving was 8.3 × 10^4^ cfu, and the mean exposure of *Salmonella *in salads per serving was 1.2 × 10^3^ cfu.

### 3.5. Food Handling, Preparation, and Storage Conditions

Results of the demographic studies revealed that 70% of the street food outlets were located in near vicinity of the road and 15% of the outlets were located near municipal garbage bin. Only seven percent of the outlets were using refrigerators to store the poultry before cooking. Sixty-five percent of the shops were using clean and dried knife for cutting and chopping. Fifty-seven percent of the outlets were using wet, unclean serving plates. Forty percent of the outlets were using cleaned serving plates with butter paper. Vegetables were not peeled and cleaned in 40% of the outlets. Eighty percent of the vendors were using dirty cloth for cleaning purpose. Ninety percent of the vendors were not using hand gloves while preparing and serving street food, and most of the vendors were using municipal tap water for drinking and utensil cleaning.

## 4. Discussion

The study demonstrated the contamination of the popularly sold street foods by either one or a combination of pathogens. *B. cereus *and* S. aureus *were the major pathogens found in poultry street foods. Microbiological hazard identification of food prepared and served in rural households of Lungwena, Malawi, indicated the incidence of *E.coli, E.coli* 0157:H7, *S.aureus*, and *Salmonella *spp. [9]. In general the study found chicken fried rice to be the most contaminated food category. This is the most common food that is served in the streets of Hyderabad.

Statistical analysis (Mann-Whitney *U* nonparametric test) was performed, and the results obtained on above parameters which indicated that Circles 1 and 5 were significantly (*P* < 0.05) different from all other circles on account of storage conditions. Table 1 gives the mean values of different pathogens in various food stuffs collected from the selected circles. *S. aureus* exists in different circles uniformly without any significant difference for chicken fried rice, chicken noodles, noodles, and rice except for chicken noodles in 1, 3, and 7 circles but in noodles circle 1 is different from 4, 6, and 7 circles, whereas circle 2 is different from 4, 6, and 7. A significant difference was observed in *B. cereus* count isolated from chicken fried rice of circles 1 (*P* < 0.001), 2, 3, 4, 5, 6, and 7. The *B. cereus* count was more in circle 1 (4.6 log cfu/g) when compared with circles 2 (1.9 log cfu/g), 3 (3.4 log cfu/g), and 4 (3.1 log cfu/g). *Salmonella* presence in different food of various circles is quite similar. The pathogenic bacterial population was significantly higher in circle 1 because demographically this circle belongs to the old part of the city wherein the density of the population is high and most of the population belonged to the lower income group; due to this, difficulties in meeting good hygienic standards were observed.

The boiled noodles and boiled rice were prepared in advance and stored at room temperature for 1–4 hrs before consumption, and this practice could have allowed the pathogens to grow to large numbers. During preparation of the raw materials at the vending site, it was not surprising that these bacterial groups were predominant in all food types. During preparation of the food, the vendor invariably left raw materials uncovered on tables which resulted in exposure to dust and possibly containing bacterial cells and spores [10, 11]. *S. aureus* detected in food could possibly have originated from vendors hands while he was cooking the food. The incidence of *B. cereus* in foods was ascribed to its presence in the raw materials in spore form which survived cooking, possibly coupled to its reintroduction into the food through postcooking contamination. The presence of *B. cereus* in the foods analysed here was of great significance since this organism produces heat sensitive and heat stable toxins associated with food poisoning [12]. Hands of the vendor and surfaces of utensils and equipment that touch raw poultry pick up pathogens from poultry surfaces and associated fluids. Cutting boards, knives, sharpeners, storage containers, and other utensils become contaminated when raw poultry is prepared [13].

 Street food outlets in Hyderabad are located at public places. The foods were served in dirty utensils. Similar observations were reported in Africa by Mosupye and Von Holy [14], Ekanem [15], Umoh and Odoba [16] and in Brazil by Hanashiro et al. [17]. In most of the food samples *Salmonella *spp. was not detected. In Hyderabad, poultry-based Street foods are served to the public along with salads (pieces of onion and lemon). *Salmonella *contamination was found more in salads than in the street food. It was found that *Salmonella *spp. is present in salads (3.2 log cfu/g) and hand washings of the food handler (3.5 log cfu/g). The study conducted on microbial evaluation of minimally processed vegetables indicated that the common pathogenic microorganisms transmitted to human beings through these products are *L. monocytogenes, E.coli* 0157:H7, and *Salmonella *spp. [18]. In Hyderabad, the salads are served along with the street foods like chicken fried rice and chicken noodles. *Salmonella* contamination which was more in salads is likely to contribute to contamination in chicken fried rice and chicken noodles although these foods by themselves did not harbor these microbes.

Forty-one percent of the vendors were using unclean and unpeeled vegetables. Previous studies have demonstrated that *Salmonella* cross-contamination occurs frequently through the use of contaminated vegetables that are improperly cleaned and undisinfected [19–21]. The serving stage is a critical point in the street food vending. Poor personal hygiene often facilitates transmission of pathogens via food to humans [22]. Most of the vendors were not using hand gloves while preparing and serving street food. Vendors were carriers of a variety of bacterial enteropathogens, including *S.typhimurium* [23]. Similarly *E. coli* was detected in hand washings of high-income and low-income mothers in India at levels of 7.0 ± 4.2 log 10 cfu/mL and 9.0 ± 5.7 log 10 cfu/mL, respectively [24].

In this study, 80% of the vendors were using dirty cloth for cleaning purpose. Cloths and sponges become contaminated when they were used to wipe drippage and smears from pieces of poultry. Once on damp cloths and sponges in association with poultry-associated drip water, the adsorbed microorganism may multiply with time. Afterwards the cloth and sponges serve as sources for further spread of pathogens to hands of the user, to the surfaces wiped, and then to many articles [25]. Most of the street food vendors cleaned their utensils with stored municipal tap water before serving the food. Previous studies demonstrated that water gets contaminated in the household during storage [26]. Pathogens can be transferred to food from utensils that are not properly cleaned with contaminated water [27, 28]. Therefore the water might have contaminated the utensils during cleaning and then cross-contaminated the food, as revealed by high incidence of pathogens in the street food.

Only 7% of the vendors were using refrigerators to store the poultry before cooking. Defective storage temperature for poultry products and prolonged holding at ambient temperatures affect microbiological quality and safety of the street food [22]. During collection of samples the environmental temperature was between 32 and 38°C. The high bacterial load of chicken fried rice and a chicken noodle was attributed to the ingredients like boiled rice and noodles kept for long time at ambient temperature. It has been reported that the infective dose for most food poisoning gram negative organisms is within the range of 10^6^ to 10^8^ cfu/g  (mL) [29]. The presence of *S.aureus* in food is associated with contamination that has been directly introduced into the food by food handlers through coughing and sneezing as well as storage of food at high temperature. Similar kind of association in food was observed by Jay [30], Kaneko et al. [31], and Sandel and McKillip [32].

In conclusion, this study found that poultry-based street foods in Hyderabad are contaminated with pathogenic bacteria like *S. aureus* and *B. cereus*. Bacterial contamination was high in salads and in drinking water. *Salmonella *contamination was more in salads than in street foods. In general chicken fried rice is the most contaminated food among poultry-based street foods in Hyderabad. Although food is cooked at a temperature high enough to inactivate bacterial pathogens, postcontamination and cross-contamination that is being promoted by unhygienic food handling and incorrect storage practices are making the safely prepared food to be unsafe. There is a need to give food safety education to the street food vendors.

## Figures and Tables

**Table 1:** Incidence of foodborne pathogens in poultry products sold in various localities of Hyderabad.

Food categories	Pathogens			Mean (Percent)
	*Staphylococcus* spp.	*Bacillus cereus*	*Salmonella *spp.	
Chicken fried rice (*N* = 94)	22 (23.4%)	53 (56.4%)	ND	39.9
Chicken noodles (*N* = 94)	23 (24.5%)	56 (59.6%)	1 (1.1%)	28.4
Plain noodles (*N* = 94)	40 (42.6%)	62 (66%)	8 (8.5%)	39
Plain rice (*N* = 94)	33 (35.1%)	48 (51.1%)	6 (6.4%)	30.9

**Table 2:** Mean concentration ranges of foodborne pathogens in various samples.

Food category	Range of microbial counts (log 10 cfu/g)			Mean
	*S.aureus*	*B.cereus*	*Salmonella *spp.	
Chicken fried rice	2.3–4.4 (3.15)^a^	2.3–4.5 (3.40)^a^	ND	3.39
Chicken noodles	2–4.1 (3.40)^a^	2–4.5 (3.62)^a^	2.6 (2.6)^a^	3.31
Boiled noodles	2.3–4.40 (3.68)^a^	2.0–4.48 (3.41)^a^	2.0–3.72 (2.63)^a^	3.53
Boiled rice	2.3–4.48 (3.38)^a^	2.0–4.48 (3.41)^a^	2.0–3.72 (2.50)^a^	3.33
Salads	2.0–4.48 (3.33)^a^	2.3–4.48 (3.30)^a^	2.0–4.18 (2.50)^a^	3.22
Drinking water	2.78–4.48 (4.40)^a^	2.3.3 (2.90)^a^	2–3.9 (3.18)^a^	3.08
Hand washings	3.15–4.48 (4.0)^a^	2.3–4.3 (3.78)^a^	2–4.3 (3.70)^a^	3.69

^a^Median concentration for food category.

**Table 3:** Microbiological quality of salads, drinking water and hand washings.

Sample category	Pathogens			Mean (Percent)
	*Staphylococcus* spp.	*Bacillus cereus*	*Salmonella *spp.	
Drinking water (*N* = 25)	7 (28%)	9 (36%)	3 (12%)	25.3
Hand washings (*N* = 17)	14 (82.4%)	9 (52.9%)	7 (41.2%)	58.8
Salads (*N* = 23)	14 (60.9%)	11 (47.8%)	10 (43.5%)	50.7
